# *In situ* determination and matching of the refractive index of the human cornea to improve polarization-resolved SHG imaging in depth

**DOI:** 10.1364/BOE.564209

**Published:** 2025-07-18

**Authors:** Poncia Nyembo Kasongo, Pierre Mahou, Jean-Marc Sintès, Gaël Latour, Marie-Claire Schanne-Klein

**Affiliations:** 1 Laboratory for Optics and Biosciences (LOB), École Polytechnique, CNRS, Inserm, Institut Polytechnique de Paris, 91120 Palaiseau, France; 2 Université Paris-Saclay, 91190 Gif-sur-Yvette, France

## Abstract

The human cornea is a highly organized tissue, which comprises hundreds of 1-3 µm thick stacked collagen lamellae. However, this microstructure is poorly characterized and requires further investigation. Polarization-resolved second harmonic generation (pSHG) microscopy is a powerful technique for this purpose because of its specificity for collagen and its sensitivity to its orientation. However, pSHG is prone to spatial resolution degradation with depth unless the immersion refractive index is matched to that of the sample, which is critical for corneas that are approximately 600 µm thick. In the absence of experimental data on the refractive index along the entire cornea, we propose a measurement method that applies to the entire cornea directly under the microscope objective. We then use an iodixanol solution to match the refractive index of the immersion medium to that of the cornea. Finally, we carefully characterize the pSHG orientation data obtained under these optimal conditions, and we show that they provide a better resolution along the entire thickness of the cornea and a better determination of the lamellae orientation.

## 1. Introduction

The human cornea is the outermost part of the eye and has unique physiological properties, namely transparency and refraction [1]. These properties are associated with the highly organized structure of the stroma, which makes up 90% of the corneal thickness [2,3]. The stroma is composed of a few hundreds 1-3 µm thick lamellae that are stacked parallel to the corneal surface and oriented mainly along the inferior-superior and the nasal-temporal axes. They are composed of well-aligned and regularly packed thin collagen fibrils (approximately 30 nm in diameter) surrounded by a nonfibrillar matrix composed of proteoglycans, glycosaminoglycans and other biomolecules in water [1]. This lamellar structure is a key factor in maintaining the corneal physiological curvature in order to ensure a constant refractive power despite daily variations in the intraocular pressure and applications of external forces. It may be affected during ocular pathologies such as keratoconus, and vision is then strongly impaired [4]. Consequently, there is a compelling need for *in situ* mapping of the collagen lamellae along the full thickness of the cornea.

Second-harmonic generation (SHG) microscopy has proven to be an effective three-dimensional (3D) imaging technique to this end [5,6]. This multiphoton modality is highly specific for fibrillar collagen without any labelling and can be easily combined with 2-photon excited fluorescence (2PEF) to visualize the corneal cellular components [7–16]. Combination with polarimetry further enhances the capability of SHG imaging since it provides the fibril orientation in every voxel, without any image processing [17–19]. This is particularly relevant for epi-imaging of corneas mounted in an opaque chamber because the small coherence length in the epi-direction results in a quasi-homogeneous image with no clear indication of the fibrils’ orientation [20]. Polarization-resolved SHG (pSHG) imaging of the collagen lamellae all along the thickness of human corneas has thus been recently reported [21]. However, the human cornea is a thick tissue (around 550 µm) that is prone to optical aberrations in depth [22–24]. In particular, the spherical aberration can deteriorate the axial resolution in depth when the refractive index of the immersion medium is not perfectly matched to the refractive index of the cornea [25]. The effect of refractive index mismatch on the optical resolution is well referenced in the confocal and multiphoton microscopy literature [25], but its effect on pSHG imaging and collagen orientation determination has not been explored in a comprehensive way. We expect that refractive index mismatch is detrimental for efficient imaging of the stromal lamellae since the thickness of these collagen lamellae is 0.2 to 2.5 µm, close to the nominal axial resolution of high numerical aperture (NA) objectives [1,26]. Any degradation of the axial resolution results in a focal volume extending over several lamellae with different orientations of the collagen fibrils. In such a case, the pSHG response is affected and no reliable orientation can be extracted from these data since their theoretical analysis assumes a homogeneous material with a well-defined mean orientation.

A first method to address this issue is to use adaptive optics to correct the aberrations [24,27]. This is a very efficient method, but it is expensive and above all, it is difficult to implement since it requires measuring and adjusting the correction at each depth. A second method is to slightly compromise the optical resolution in the anterior part of the cornea in order to limit its degradation in the posterior stroma. This is done by using water or Lacrigel as the immersion medium and adjusting the objective correction collar to obtain the larger SHG signal in the middle of the stroma. This a cheap and simple method, but its efficiency is moderate and many pixels in the posterior stroma have to be filtered out due to a low quality pSHG response [21].

In this article, we propose an effective and yet inexpensive method that basically consists of using an immersion medium with the same refractive index as the one of the cornea and adjusting the objective correction collar to this value. The main difficulty with this optimal method is the accurate determination of the refractive index of the human cornea. Indeed, only few articles in the literature report measurements or calculations of the corneal refractive index, which is moreover heterogenous along the different corneal layers, so there are no clear experimental data on the mean refractive index along the entire stroma [2,28–30]. Furthermore, this refractive index may vary from one cornea to another and it may also be affected by the fixation prior to imaging. We therefore propose a method to accurately measure the mean refractive index of the entire cornea. We then use an iodixanol solution [31] to adjust the refractive index of the immersion medium to the same value. Comparison of the pSHG data acquired under these optimal conditions with the previous ones based on the resolution compromises shows that this is an efficient method that provides a better resolution along the full thickness of the cornea and allows a reliable determination of the collagen orientation in more pixels.

## 2. Materials and methods

### 2.1. Human cornea samples

This study was conducted in accordance with the tenets of the Declaration of Helsinki and followed international ethical requirements for human tissues. Handling of human corneas was declared to the French administration (CODECOH agreement DC-2018-3300). Human corneas, which were unsuitable for transplantation and authorized for scientific use by the donor family, were obtained from the French Eye Bank (Banque Française des Yeux, BFY, Paris). Eight corneas were processed as reported previously [21]: they were preserved in an appropriate culture medium (Stemalpha 2, Eurobio) in an incubator set at 31°C, then placed for 48 h in Stemalpha 3 without phenol red to induce deswelling and elimination of the phenol red present in the previous culture medium, prior to fixation in 4% paraformaldehyde (PFA) for 24 h at 4 °C. These fixed corneas were then stored in 1% PFA at 4 °C until imaging. Detailed information about these corneas is listed in the Supplement 1 Table ST1.

**Fig. 1.**
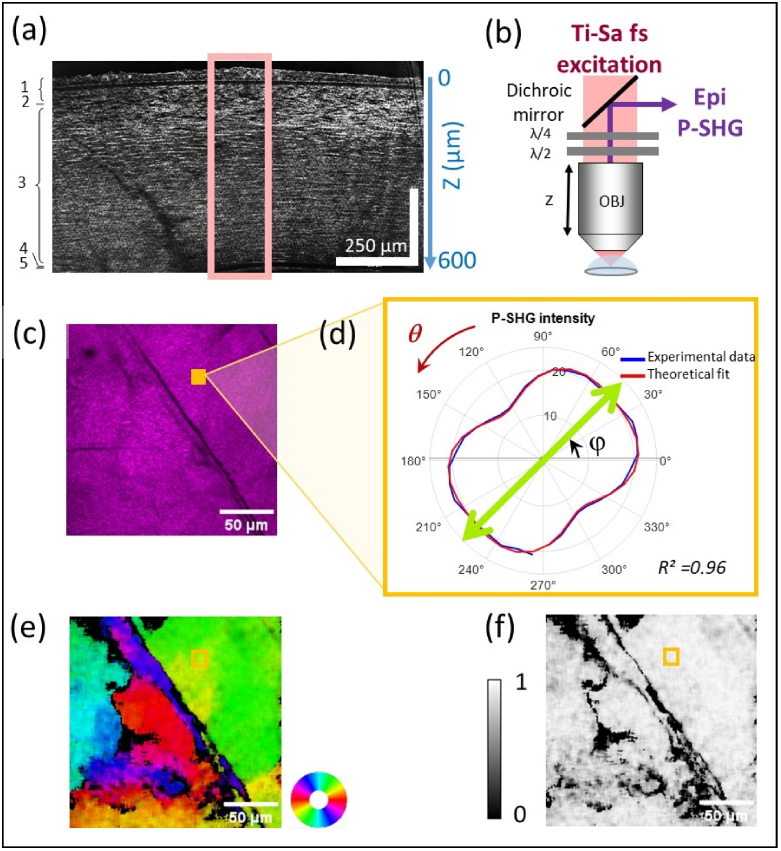
Experimental setup and P-SHG data analysis. (a) LC-OCT transverse image of a human cornea (#1 in Table 1). The pink rectangle at the corneal apex corresponds approximately to the region where the *en face* pSHG image stack is recorded. 1- Epithelium layer, 2- Bowman layer, 3- Corneal stroma, 4- Descemet layer, 5- Endothelial layer. (b) Scheme of the pSHG microscope using Ti-Sa fs excitation laser source, half and quarter waveplates to control the excitation polarization and an Apochromat 20X/1.0 Zeiss objective lens. (c) Epi-detected SHG image in the middle stroma (approximately 300 µm deep) obtained as the average of all images recorded with different polarization orientations of the laser excitation. Negative contrast areas correspond to areas without fibrillar collagen or to stromal striae. (d) Polarimetric diagram showing the variation of the SHG signal on a 6 × 6 binned pixel (blue) as a function of the polarization orientation of the laser excitation. Good agreement (R^2^= 0.96) is observed with the theoretical Eq. (1) (red). (e) Orientation map in the same plane as (c). The color wheel indicates the orientation code. Dark pixels are non-valid pixels (R^2^<0.7). (f) Map of R^2^ in the same plane as (c). Scale bar: black = 0 and white = 1.

All the corneas were first imaged using Line-field Confocal Optical Coherence Tomography (LC-OCT, Damae Medical, France) [32,33] to verify the efficiency of the deswelling process (cornea thickness < 650 µm) and the absence of large structural defects, *i.e.* hyper-reflective or hypo-reflective regions larger than ≈ 0.01 mm^2^ (excluding star-like hyper-reflective regions corresponding to keratocytes) (Fig. 1(a)).

### 2.2. Cornea holder for pSHG microscopy

The corneas were then imaged on the pSHG microscope. The nasal–temporal and superior–inferior physiological axes of the corneal button were visually identified and oriented along the X or Y axis in the imaging plane. Imaging was performed in the apex region to ensure that the corneal surface was parallel to the imaging plane and to minimize the spherical aberration.

**Fig. 2.**
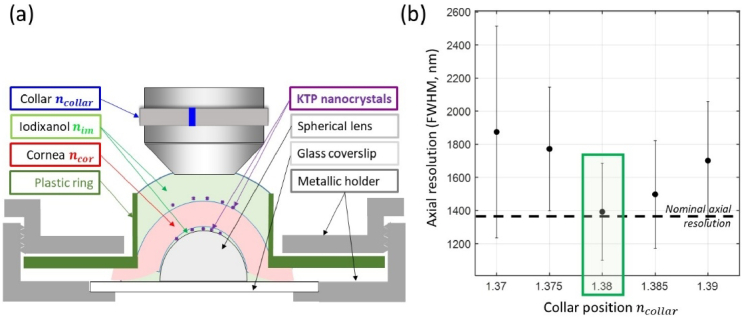
Measurement of cornea refractive index. (a) Cornea mounting under the objective lens with collar set to $n_{collar}$ (Apochromat 20X/1.0, Zeiss). The cornea is immersed on both sides in an iodixanol solution with a refractive index $n_{im}$ . It is clamped on top of a spherical lens to maintain cornea physiological curvature. KTP nanocrystals are deposited on the cornea anterior surface and on the spherical lens for PSF measurements. (b) Axial resolution measured under the cornea #1 in Table 1 as a function of the objective correction collar position. Here $n_{im}$ is fixed to 1.38. The minimum axial resolution is compared to the nominal one measured with water immersion on a glass slide (black broken line). Bars correspond to the standard deviation of the measurements in all the KTP nanocrystals in the field of view (see Sup. Fig. S2(c)).

A specific cornea holder was designed to maintain the physiological curvature of the cornea and to enable the use of a non-viscous immersion medium (Fig. 2(a)). The cornea was positioned on a small plano-convex lens with a diameter and radius of curvature identical to its own and the sclera was clamped by a plastic ring maintained by a metallic screwed holder as reported previously [21]. This plastic ring comprised a 4 mm-high polylactic acid collar affixed around the cornea, creating a sealed perimeter to be filled by the immersion medium (Fig. 2(a)).

### 2.3. Immersion medium

The refractive index of the immersion medium was tuned by using various concentrations of Optiprep (OptiPrep Density Gradient Medium, D1556, Sigma-Aldrich) in Phosphate-buffered saline (PBS) solution. Optiprep is a commercial iodixanol 60% stock solution that displays a high refractive index of 1.429 and is transparent in the visible and near-IR range. It has been shown to be non toxic and enable index-matched imaging of live specimens [31]. The variation of the refractive index as a function of the Optiprep concentration in PBS was calibrated in a series of different dilutions using a refractometer at 589 nm (Refracto30PX, Mettler-Toledo, Switzerland) (Sup. Fig. S1). The refractive index of every immersion solution was systematically measured before pSHG imaging at the same temperature (21°).

### 2.4. pSHG imaging

SHG imaging is performed on a custom-built upright laser-scanning multiphoton microscope equipped with a Titanium-Sapphire (Ti-Sa) laser set at 860 nm (Mai-Tai, Spectra-Physics) as previously reported [21] (Fig. 1(b)). A 20x, 1.0 Numerical Aperture (NA) water-immersion objective lens with a correction collar (W Plan-Apochromat 20×/1.0 Korr DIC, Zeiss) is used in order to enable adjustment to a refractive index of 1.32 to 1.40 (Sup. Fig. S2(a)). The axial and lateral resolutions (Full Width at Half Maximum) are measured as 0.35 µm × 1.36 µm on a glass slide with water immersion (see below) and the full Field of View (FOV) is 634 × 634 µm^2^. Multiphoton signals are detected in the backward direction using photon-counting photomultiplier tubes (P25PC, Electron Tubes, UK). A dichroic mirror (FF-458-Di01, Semrock) is used to separate SHG from 2PEF signals and appropriate spectral filters are used to reject laser excitation (FF01-680/SP and FF01-720/SP, Semrock) and select SHG signal (FF01-427/10 interferential filter, Semrock).

Polarization-resolved SHG imaging is performed by inserting a quarter and a half achromatic wave-plates at the back pupil of the objective lens. The quarter waveplate corrects for the ellipticity introduced by the optical components in the microscope in order to have a perfectly linear polarization (ellipticity less than 4.4%) in a reduced field of view of 200 × 200 µm^2^, whereas the half waveplate enables the rotation of the polarization orientation. Series of 18 SHG images are then recorded sequentially while rotating the linear polarization orientation from 0° to 170° with 10° steps.

Z-stacks of these pSHG images are recorded along the full cornea thickness at a pixel rate of 200 kHz and with a voxel size of 1.0 µm in XY directions and 2.0 µm in Z direction. The excitation power at the focus is linearly increased in depth to compensate for SHG signal attenuation, starting from 50 mW at the anterior surface (Z = 0) to 130 mW at the posterior surface (Z ≈ 600 µm). We verified that no tissue damage occurred in this range of power by recording sequentially two Z-stacks of pSHG images and checking that they were similar.

### 2.5. pSHG theoretical analysis and image processing

The SHG response of collagen is described theoretically by a second-order susceptibility tensor $\chi^{\left(2\right)}$ . It is assumed to have a cylindrical symmetry with only two independent components in the Kleinman’s approximation [17]. This tensorial formalism shows that the SHG signal varies with the orientation of the collagen fibrils *φ* relative to the orientation of the incident field *θ* within the imaging plane. Polarization-resolved SHG is thus given theoretically by the following equation:

(1) $${\text{I}}_{SHG}^{th}(\theta )=a_{0}+a_{2}\;cos[2(\theta -\varphi )]+a_{4}\;cos[4(\theta -\varphi )]$$

The parameters $a_{0},a_{2}$ and $a_{4}$ depend on the susceptibility tensor components and on various geometrical and experimental parameters, including the square of the incident laser intensity.

To process pSHG data in a faster way, we use a Fast Fourier Transform (FFT) of our angular data ${\text{I}}_{SHG}^{exp}(\theta )$ in every pixel (Fig. 1(d)). The phase of the Fourier components at 2θ
 and 4θ
 then provides the collagen orientation *φ* [19,34]. We also assess the reliability of this orientation measurement by computing a coefficient of determination *R^2^*. Indeed, the FFT of the experimental data ${\text{I}}_{SHG}^{exp}(\theta )$ can exhibit components at other harmonics than 2θ
 and 4θ
 , which means that this experimental data does not obey the theoretical Eq. (1). This coefficient of determination *R^2^* is calculated as:

(2) $$R^{2}=\max\left(0,1-\frac{\sum_{\theta }{\left[I_{SHG}^{\exp}(\theta )-I_{FFT}(\theta )\right]}^{2}}{\sum_{\theta }{\left[I_{SHG}^{\exp}(\theta )-\left\langle I_{SHG}^{\exp}(\theta )\right\rangle \right]}^{2}}\right)$$

where

(3) IFFT(θ
)=α
0+α
2e2iθ
+α
4e4iθ
+c.c.

Here α
0=a0 , α
2=a2e−
2iφ
2 and α
4=a4e−
2iφ
4 where φ
2 and φ
4 are the phases of the Fourier components at 2θ
 and 4θ
 respectively [19]. A low value of *R^2^* is obtained when the FFT of the experimental data contains other Fourier components than the ones used to calculate $I_{FFT}(\theta )$ . We then set a threshold at *R^2^* *=* *0.7* and filter out all the voxels with *R^2^* *<* *0.7.* This value is empirically chosen to get a sufficient number of valid voxels, while filtering out incorrect angular measurements; we have checked that it enables to get orientation maps that show consistent angles in lamellar domains. We finally obtained 3 maps:

• The SHG intensity map obtained as the average of all ${\text{I}}_{SHG}^{exp}(\theta )$ images (that is $a_{0}$ ), which is similar to a SHG image acquired with circular excitation and same total acquisition time (Fig. 1(c)).
• The *φ* orientation map, which is depicted using HSV look-up table: the Hue codes the orientation, S = 1 and the Brightness V codes the *R^2^.* Voxels that have been eliminated because of *R^2^* *<* *0.7* are depicted in black (Fig. 1(e)).
• The *R^2^* map (Fig. 1(f)).

In order to get a higher signal to noise ratio and obtain more valid voxels with $R^{2}\geq 0.7$ , all pSHG images are preprocessed using a 6 × 6 binning filter. This yields an effective resolution of 6 µm in the lateral direction. This resolution is smaller than the size of the lamellar domains in the cornea, ensuring a homogenous pSHG response within the binned voxel.

### 2.6. Resolution measurements

The Point Spread Function (PSF) is measured using KTiOPO_4_ (KTP) nanocrystals 80-100 nm in diameter as recently reported [35]. KTP nanocrystals are very bright SHG nano-emitters with the key advantages of no photobleaching compared to fluorescent beads and of stable colloidal suspensions in water. After appropriate dilution, a drop of KTP nanocrystals solution is deposited on a glass side coated with poly-D-lysine to ensure the nanocrystals adherence. This glass side is then used to measure the optimal resolution of our setup by setting the objective correction collar to 1.33 and using water immersion. After acquisition of a Z-stack of SHG images with 300 nm axial step, a Matlab image analysis workflow is used to automatically compute the lateral and axial sizes of the PSF of all nanocrystals in the Z-stack. The axial and lateral resolutions are then calculated as the mean ± standard deviation of these distributions [35] (Sup. Fig. S2).

A similar protocol is used to measure the spatial resolution above and below the cornea in order to estimate the resolution degradation in depth. KTP nanocrystals are directly deposited on the anterior surface of the cornea after coating with poly-D-lysine. The same method lacks reproducibility for the posterior side of the cornea as it may not be perfectly flat and the signal to noise ratio is degraded by the vicinity of the strong SHG signal from the posterior stroma. To overcome these issues, the KTP nanocrystals are deposited on the glass lens placed under the cornea in the cornea chamber. This glass lens is beforehand coated with poly-D-lysine and immersed in the same immersion medium as the one used between the objective lens and the cornea (Fig. 2(a)) in order to ensure optical contact with continuous refractive index and not degrade the measured resolution.

## 3. Measurements of the refractive index of the human cornea

### 3.1. Experimental protocol

Refractive index measurements are a complex issue in thick tissues because commercial refractometers are designed for liquid samples or thin solid ones. We have thus designed a protocol based on our multiphoton microscope that is specifically dedicated to thick transparent tissues. This protocol relies on PSF measurements before and after propagation through the tissue. The measured axial resolutions are minimized in these two locations if the refractive index $n_{im}$ of the immersion medium is equal to the mean refractive index $n_{cor}$ of the tissue and the objective correction collar is set to this shared value. This configuration indeed minimizes the spherical aberration, which would degrade the axial resolution in depth. When $n_{cor}\neq n_{im}$ , the axial resolution is optimized in depth when the optical phases with and without the sample are equal [36]. It corresponds to the following position $n_{collar}$ of the objective correction collar [37]:

(4) $$n_{collar}WD=n_{cor}e_{cor}+\;n_{im}(WD-\;e_{cor})$$

Here $e_{cor}$ is the depth of the measurement within the tissue and WD is the objective working distance. The Eq. (4) enables to calculate the refractive index $n_{cor}$ of the tissue:

(5) $$n_{cor}=\frac{WD}{e_{cor}}n_{collar}+\frac{e_{cor}-WD}{e_{cor}}n_{im}$$

The refractive index $n_{im}$ of the immersion medium can be tuned by using various concentrations of Optiprep [31] and is measured using a commercial refractometer. The tissue, here the cornea, is mounted with this immersion medium under the objective, after KTP nanocrystals have been deposited on its anterior surface and under the endothelium, on the spherical holder, as shown in Fig. 2(a).

Our protocol is then organized in three steps:

1. The refractive index $n_{im}$ of the immersion medium is set to an arbitrary value close to the expected one for the cornea and a series of PSF measurements are performed under the cornea when tuning the position of the objective correction collar $n_{collar}$ around $n_{im}$ (Fig. 2(b)).
2. The correction collar position $n_{collar}$ corresponding to the minimal axial resolution obtained in step 1 is used to calculate the refractive index $n_{cor}$ of the cornea using Eq. (5).
3. A new immersion medium is prepared at the refractive index $n_{im}=\;n_{cor}$ determined in step 2, and a new series of PSF measurements are performed under the cornea when tuning the position of the objective correction collar $n_{collar}$ around $n_{im}$ . We expect that the PSF is minimized at $n_{collar}=n_{im}$ and that it is close to the value at the top of the cornea, which means that the refractive index $n_{cor}$ of the cornea has been correctly determined in the previous steps.

### 3.2. Refractive index of fixed human corneas

Refractive index measurements according to our protocol were performed on 8 fixed human corneas. Table 1 shows that these measurements are highly reproducible among the 8 corneas and yield a refractive index of 1.380 ± 0.005. The minimum axial resolution measured under the cornea shows more variation. This is attributed to residual aberrations associated with tissue heterogeneities, which can vary widely from one cornea to another. Nevertheless, most of the minimal axial resolutions are close to the nominal one (1.36 µm), which means that these corneas are well preserved and that aberrations are minimized.

**Table 1.** Measurements of the refractive index $n_{cor}$ of 8 fixed corneas

Cornea Number	Thickness [µm]	Optimal n_collar_	n_im_	Minimal axial PSF [µm]^*a*^	n_cor_ (± 0.005)
1	610	1.38	1.379	1.36 ± 0.29	1.380
2	480	1.38	1.379	1.50 ± 0.30	1.380
3	600	1.38	1.381	1.36 ± 0.20	1.380
4	450	1.38	1.381	1.34 ± 0.10	1.380
5	430	1.38	1.380	1.32 ± 0.15	1.380
6	540	1.38	1.379	1.59 ± 0.28	1.380
7	570	1.38	1.380	1.37 ± 0.17	1.380
8	550	1.38	1.380	1.33 ± 0.29	1.380

^*a*^ The minimal axial resolution measured under the cornea in the optimal condition $n_{cor}=n_{im}=n_{collar}$ .

## 4. p-SHG imaging of human cornea in depth

In our previous report of pSHG imaging of human corneas [21], an ophthalmic gel (Lacrigel, Europhta), with a refractive index of 1.33, was used as immersion medium to avoid cornea dehydration. To reduce the spherical aberration and avoid low contrast in the posterior stroma, the objective correction collar was set at 1.36 to maximize the SHG signal at half the depth of each cornea (typically 250 µm) at the expense of a degraded resolution at the top and bottom of the sample. Therefore, to evaluate the benefit of index matching on corneal pSHG imaging in depth, we compare the new index-matched configuration with this previous one and with the basic configuration with the objective correction collar tuned to the Lacrigel refractive index.

**Fig. 3.**
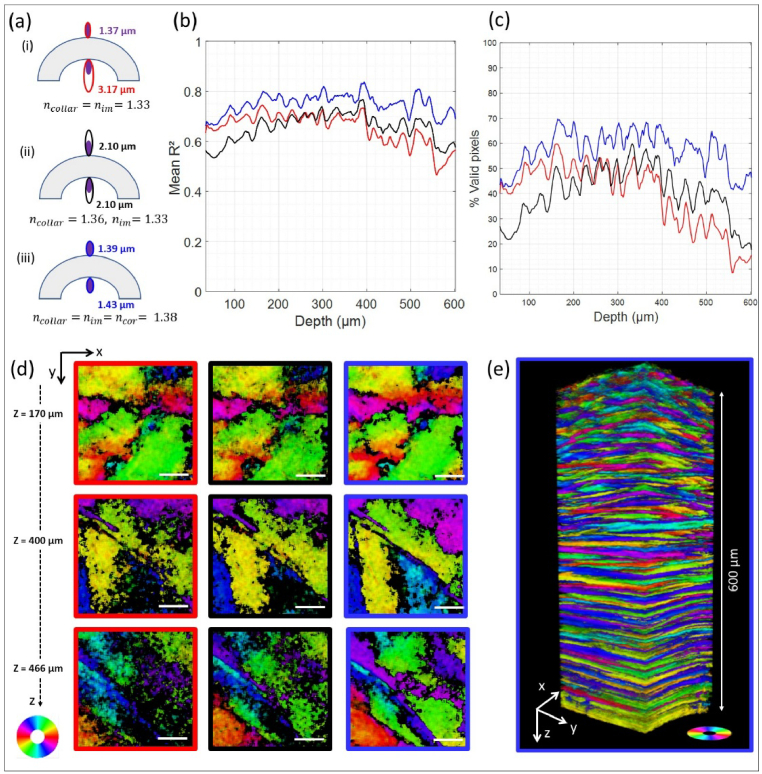
pSHG imaging of human cornea #1 without or with index-matching. (a) Axial resolutions above and under the cornea for the 3 imaging configurations: (i) $n_{collar}=\;n_{im}=1.33$ , (ii) $n_{collar}=\;1.36,\;\;n_{im}=1.33$ and (iii) $n_{collar}=\;n_{im}=1.38$ ; (b) Mean R^2^ and (c) percentage of valid pixels after applying the same threshold (R^2^>0.7) as a function of depth for the 3 imaging configurations: (i) red solid line, (ii) black solid line, (iii) blue solid line; (d) Comparison of XY images at 3 different depths for the 3 imaging configurations: (i) red rectangle, (ii) black rectangle, (iii) blue rectangle ; Scale bar: 50 µm ; The orientation is displayed using HSV code: Hue is the orientation as shown in the color wheel, S = 1 and V = R^2^ (e) 3D reconstruction of corneal lamellar organization from P-SHG imaging with index-matching (configuration (iii), 193 × 193 × 600 µm^3^).

Here are the 3 configurations studied and the axial resolutions measured above and under the cornea (Fig. 3(a)):

(i) **Without index-matching**, $n_{collar}=n_{im}=1.33,$ using Lacrigel as an immersion medium. In this basic configuration, the axial resolution is the nominal one (1.37 ± 0.03 µm) on top of the cornea, but it is strongly degraded under the cornea (3.17 ± 0.03 µm).
(ii) **Without index-matching**, $n_{collar}=1.36,n_{im}=1.33,$ using Lacrigel as an immersion medium as in [21]. In this intermediate configuration, the axial resolution is similarly degraded on both sides on the cornea (2.10 ± 0.03 µm)
(iii) **With index-matching**, $n_{collar}=n_{im}=n_{cor}=1.38$ , using iodixanol solution as an immersion medium. This configuration yields the better axial resolution under the cornea (1.43 ± 0.03 µm), close to the nominal one observed on top of the cornea (1.39 ± 0.03 µm).

It should be noted that none of these 3 configurations eliminates scattering, which slightly reduces the SHG signal with depth. This means that it is necessary to compensate for scattering effects by increasing the excitation power with depth even for the refractive index-matched configuration. However, using the same rate of increase in excitation power with depth, the SHG signal is higher for the refractive index-matched configuration (Sup Fig S3).

The first criterion for comparing the three configurations studied is the average value in each Z-plane of the coefficient of determination R^2^ along the depth of the cornea (Fig. 3(b)). In the configuration (i), the mean R^2^ decreases with depth as expected (red curve). Indeed, the axial size of the focal volume increases with depth and may then extend over 2 sequential lamellae with different orientations, which distorts the pSHG response and results in a low R^2^. In the configuration (ii), the mean R^2^ is lower in the anterior stroma and higher in the posterior stroma (black curve) than in the configuration (i), as expected from the resolution measurements. Finally, in the configuration (iii), i.e. with refractive index-matching, the mean R^2^ is higher than in the other configurations all along the stroma (blue curve). However, the mean R^2^ is slightly smaller in the anterior stroma due to the thinner collagen lamellae in this region: 0.2 to 1.2 µm compared to 1 to 2.5 µm in the posterior stroma [26]. Thus, even in the optimal configuration corresponding to an axial resolution of 1.38 µm, the focal volume may include 2 consecutive lamellae with different orientations.

The coefficient of determination is used to filter out pixels with R^2^<0.7. These pixels are considered as invalid for pSHG analyses and depicted in black. The second criteria to evaluate the benefit of index-matching is thus the percentage of valid pixel in depth as displayed in Fig. 3(c). As expected from the R^2^ behavior, the number of valid pixels strongly decreases in depth for the configuration (i). The decrease of valid pixels in depth is lower for the configuration (ii), at the expense of a lower number in the anterior part where the axial resolution is slightly degraded. Finally, the number of valid pixels is larger all along the cornea depth with the index-matching configuration. This improvement is observed for any value of the R^2^ threshold (Sup Fig S4). It is particularly significant in the posterior stroma, where it reaches a factor 2 for a R^2^ threshold of 0.7 (Sup Fig S4). It enables to get around the same number of valid pixels all along the corneal stroma, instead of having less valid pixels in depth as in the other configurations. However, the number of valid pixels is slightly lower in the anterior stroma, consistent with the lower R^2^ values observed in this region with thin lamellae.

Accordingly, 2D orientation maps extracted from the pSHG data show a low number of black pixels throughout the depth (600 µm) of the cornea (Fig. 3(d)). This is clearly seen in depth, where the configurations without index matching show no orientation determination in many pixels, especially the basic configuration. In contrast, index matching allows robust orientation determination in most pixels and clear visualization of the lamellar domains with similar orientation. This is also observed in the 3D reconstruction of the pSHG orientation maps as shown in Fig. 3(e): it shows good quality throughout the entire depth of the cornea, allowing the observation of lamellar domains with alternating orientations.

The other corneas under study show similar results as shown in the Supplement 1 Table ST2. The mean coefficient of determination < R^2^> and the mean number of valid voxels < Nv > in the posterior half of the corneas are larger in the configuration with index matching (iii) than in the other configurations (ii) without index matching: <R^2^> = 0.74 and < Nv >  = 55% for the configuration (iii), <R^2^> = 0.67 and < Nv >  = 40% for the configuration (ii) and < R^2^> = 0.69 and < Nv >  = 45% for the configuration (i).

## 5. Discussion

In this paper, we have developed a method to measure the mean refractive index of fixed human corneas directly under the microscope objective. This method provides the same value in 8 fixed human corneas: n = 1.380 ± 0.005. We attribute this constant result to the PFA fixation, which homogenizes the tissue density. We assume that the refractive index in a fixed cornea is more homogeneous than in an in vivo cornea [38], and that it doesn’t vary significantly between different fixed corneas. The precision of this measurement is however limited by the manual rotation of the objective correction collar. It could be improved by using a motorized correction collar, which would also allow to automate the refractive index measurement [39]. Nevertheless, the current precision allows an optimal axial resolution in depth and is therefore sufficient for optimal pSHG imaging in depth, which is the main purpose of this study.

In the context of pSHG imaging, our measurement method could be generalized to any thick tissue, provided that it is transparent enough to allow trans-detection of multiphoton signals. In particular, it could be used to verify the refractive index of cleared tissues. Note however that this method also requires a low tissue birefringence as it is the case for *en face* imaging of cornea [30,40]. In contrast, the presence of birefringence would result in two different refractive indices along the 2 axes in the imaging plane, which could be measured with 2 different incident polarizations, but couldn’t be equaled together to the refractive index of the immersion medium and the correction collar for pSHG imaging. Birefringence is observed in tissues with a high density of collagen fibers oriented in the same direction, such as tendon [41], but it is negligible in tissues with a low density of collagen fibers or with a high density of collagen fibers with random orientations. In the cornea, the collagen lamellae are birefringent, but they are equally distributed along 2 main perpendicular orientations, so that the overall birefringence of the full stroma is negligible and our method is applicable [21,30].

After measuring the refractive index of fixed corneas, we have performed pSHG imaging while adjusting to the same value (i) the refractive index of the immersion medium using an iodixanol solution of adjustable concentration and (ii) the position of the objective correction collar. This refractive index matching imaging configuration yields a better axial resolution along the entire depth of the cornea and consequently better pSHG results as shown by the higher number of voxels with reliable measurements of the collagen orientation. Refractive index matching is indeed critical for cornea pSHG imaging because the thickness of the collagen lamellae (≈ 0.2 to 2.5 µm) is close to the optimal axial resolution (≈ 1.36 µm with our 20x, 1.0 NA objective lens). When the axial resolution is degraded due to the spherical aberration, many voxels encompass 2 sequential lamellae with different orientations. There may still be a main orientation corresponding to the main lamellae within the voxel, but the presence of collagen fibrils with another orientation strongly affects the pSHG response and impedes any reliable orientation determination. Indeed, the theoretical background of pSHG assumes a cylindrical symmetry of the collagen fibril assembly within the focal volume. It means that all the fibrils must have the same orientation or only exhibit a slight disorder, with a well-defined main orientation [42–45].That is the reason why it is crucial to filter out voxels where the pSHG response is distorted as done in this study by computing a R^2^ coefficient of determination [41] or using alternative methods in other reports [46,47]. More complex fibril distributions can be taken into account by considering a trigonal symmetry instead of a cylindrical one, that is by adding another component to the second-order susceptibility tensor χ^(2)^ [48–51]. This approach has been shown to better fit the pSHG data of complex fibril assemblies and to result in a larger R^2^, but the accuracy of the orientation determination still needs to be estimated using numerical simulations. Therefore, we strongly believe that the best way to improve pSHG data fitting is to preserve the axial resolution in depth using a refractive index matching imaging configuration. These considerations may be generalized to all biological tissues where the size of the collagen fibrils assemblies with similar orientation is around a few micrometers, that is to many tissues. For instance, it applies to collagen fibers in skin dermis or in arteries.

Using this refractive index matching imaging configuration, we have recorded improved collagen orientation maps of the entire human cornea, up to 600 µm deep. It should facilitate subsequent processing aiming at segmenting collagen lamellae. These lamellae are defined as regions with similar orientations, that are depicted with similar colors in the orientation maps in Fig. 3. Less invalid black pixels should enable to determine the boundaries of these lamellae in a more accurate way. This lamellae structure could then feed mechanical models aiming at simulating the corneal mechanical properties with improved accuracy [52,53].

## 6. Conclusion

In conclusion, we have shown that *in situ* measurements of the corneal refractive index in our multiphoton setup enables improved pSHG imaging of collagen orientation based on refractive index matching. This method should prove efficient for many other collagen tissues with micrometer-sized lamellae or fibers. In the human cornea, it significantly improves the number of voxels with reliable orientation determination and thus the mapping of the lamellar structure of the corneal stroma. Furthermore, our data also show that the refractive index of fixed human corneas is homogeneous and doesn’t vary significantly between corneas. Our measurement of *n* = 1.380 ± 0.005 can therefore be used with high confidence in all fixed human corneas.

## Supplemental information

Supplement 1 Supplemental Tables and Figures https://doi.org/10.6084/m9.figshare.29529080

## Data Availability

Data underlying the results presented in this paper are not publicly available at this time but may be obtained from the authors upon reasonable request.

## Supplemental document

See Supplement 1 for supporting content.

## References

[1] Krachmer J. Mannis M. Holland E., *Cornea* - *3rd edition* (Mosby Elsevier, 2011).

[2] Maurice D. M., “The structure and the transparency of the cornea,” J. Physiol. 136(2), 263–286 (1957). 10.1113/jphysiol.1957.sp005758 13429485 PMC1358888

[3] Meek K. M. Knupp C., “Corneal structure and transparency,” Prog. Retin. Eye Res. 49, 1–16 (2015). 10.1016/j.preteyeres.2015.07.001 26145225 PMC4655862

[4] Hashemi H. Heydarian S. Hooshmand E. et al., “The Prevalence and Risk Factors for Keratoconus: A Systematic Review and Meta-Analysis,” Cornea 39(2), 263–270 (2020). 10.1097/ICO.0000000000002150 31498247

[5] Chen X. Y. Nadiarynkh O. Plotnikov S. et al., “Second harmonic generation microscopy for quantitative analysis of collagen fibrillar structure,” Nat. Protocols 7(4), 654–669 (2012). 10.1038/nprot.2012.009 22402635 PMC4337962

[6] Bancelin S. Aimé C. Gusachenko I. et al., “Determination of collagen fibril size via absolute measurements of second-harmonic generation signals,” Nat. Commun. 5(1), 4920 (2014). 10.1038/ncomms5920 25223385

[7] Yeh A. T. Nassif N. Zoumi A. et al., “Selective corneal imaging using combined second-harmonic generation and two-photon excited fluorescence,” Opt. Lett. 27(23), 2082–2084 (2002). 10.1364/ol.27.002082 18033448

[8] Han M. Giese G. Bille J. F., “Second harmonic generation imaging of collagen fibrils in cornea and sclera,” Opt. Express 13(15), 5791–5797 (2005). 10.1364/opex.13.005791 19498583

[9] Morishige N. Wahlert A. J. Kenney M. C. et al., “Second-harmonic imaging microscopy of normal human and keratoconus cornea,” Invest. Ophthalmol. Vis. Sci. 48, 1087–1094 (2007). 10.1167/Iovs.06-1177 17325150 PMC1894888

[10] Aptel F. Olivier N. Deniset-Besseau A. et al., “Multimodal nonlinear imaging of the human cornea,” *Invest. Ophthalmol. Vis. Sci* 51(5), 2459–2465 (2010). 10.1167/iovs.09-4586 20071677

[11] Park C. Y. Lee J. K. Chuck R. S., “Second Harmonic Generation Imaging Analysis of Collagen Arrangement in Human Cornea,” Invest. Ophthalmol. Vis. Sci. 56(9), 5622–5629 (2015). 10.1167/iovs.15-17129 26313297 PMC4553931

[12] Winkler M. Shoa G. Xie Y. L. et al., “Three-Dimensional Distribution of Transverse Collagen Fibers in the Anterior Human Corneal Stroma,” Invest Ophth Vis Sci 54(12), 7293–7301 (2013). 10.1167/iovs.13-13150 PMC4589141 24114547

[13] Benoit A. Latour G. Schanne-Klein M.-C. et al., “Simultaneous microstructural and mechanical characterization of human corneas at increasing pressure,” J. Mech. Behav. Biomed. Mater. 60, 93–105 (2016). 10.1016/j.jmbbm.2015.12.031 26773650

[14] Mercatelli R. Ratto F. Rossi F. et al., “Three-dimensional mapping of the orientation of collagen corneal lamellae in healthy and keratoconic human corneas using SHG microscopy,” J. Biophot. 10(1), 75–83 (2017). 10.1002/jbio.201600122 27472438

[15] Batista A. Breunig H. G. Konig A. et al., “High-resolution, label-free two-photon imaging of diseased human corneas,” J. Biomed. Opt. 23(03), 1 (2018). 10.1117/1.Jbo.23.3.036002 29500874

[16] Avila F. J. Gambin A. Artal P. et al., “In vivo two-photon microscopy of the human eye,” Sci. Rep. 9(1), 10121 (2019). 10.1038/s41598-019-46568-z 31300680 PMC6626016

[17] Stoller P. Reiser K. M. Celliers P. M. et al., “Polarization-modulated second harmonic generation in collagen,” Biophys J. 82(6), 3330–3342 (2002). 10.1016/S0006-3495(02)75673-7 12023255 PMC1302120

[18] Tiaho F. Recher G. Rouède D., “Estimation of helical angle of myosin and collagen by second harmonic generation imaging microscopy,” Opt. Express 15(19), 12286–12295 (2007). 10.1364/oe.15.012286 19547597

[19] Raoux C. Schmeltz M. Bied M. et al., “Quantitative structural imaging of keratoconic corneas using polarization-resolved SHG microscopy,” Biomed. Opt. Express 12(7), 4163–4178 (2021). 10.1364/BOE.426145 34457406 PMC8367248

[20] Latour G. Gusachenko I. Kowalczuk L. et al., “*In vivo* structural imaging of the cornea by polarization-resolved Second Harmonic microscopy,” Biomed. Opt. Express 3(1), 1 (2012). 10.1364/BOE.3.000001 22254163 PMC3255328

[21] Raoux C. Chessel A. Mahou P. et al., “Unveiling the lamellar structure of the human cornea over its full thickness using polarization-resolved SHG microscopy,” Light Sci. Appl. 12(1), 190 (2023). 10.1038/s41377-023-01224-0 37528091 PMC10394036

[22] Bueno J. M. Palacios R. Chessey M. K. et al., “Analysis of spatial lamellar distribution from adaptive-optics second harmonic generation corneal images,” Biomed. Opt. Express 4(7), 1006–1013 (2013). 10.1364/BOE.4.001006 23847727 PMC3704083

[23] Bueno J. M. Skorsetz M. Palacios R. et al., “Multiphoton imaging microscopy at deeper layers with adaptive optics control of spherical aberration,” J. Biomed. Opt. 19(1), 011007 (2013). 10.1117/1.JBO.19.1.011007 23864036

[24] Kang S. Kang P. Jeong S. et al., “High-resolution adaptive optical imaging within thick scattering media using closed-loop accumulation of single scattering,” Nat. Commun. 8(1), 2157 (2017). 10.1038/s41467-017-02117-8 29255208 PMC5735168

[25] Egner A. Hell S. W., “Aberrations in Confocal and Multi-Photon Fluorescence Microscopy Induced by Refractive Index Mismatch,” in *Handbook Of Biological Confocal Microscopy* , Pawley J. B., ed. (Springer US: Boston, MA, 2006), pp. 404–413.

[26] Komai Y. Ushiki T., “The three-dimensional organization of collagen fibrils in the human cornea and sclera,” Invest. Ophthalmol. Vis. Sci. 32, 2244–2258 (1991). 2071337

[27] Zeng J. Mahou P. Schanne-Klein M.-C. et al., “3D resolved mapping of optical aberrations in thick tissues,” Biomed. Opt. Express 3(8), 1898–1913 (2012). 10.1364/BOE.3.001898 22876353 PMC3409708

[28] Patel S. Marshall J. Fitzke F. W. 3rd, “Refractive index of the human corneal epithelium and stroma,” J. Refract Surg 11(2), 100–141 (1995). 10.3928/1081-597X-19950301-09 7634138

[29] Leonard D. W. Meek K. M., “Refractive indices of the collagen fibrils and extrafibrillar material of the corneal stroma,” Biophys. J. 72(3), 1382–1387 (1997). 10.1016/S0006-3495(97)78784-8 9138583 PMC1184520

[30] Teulon C. Gusachenko I. Latour G. et al., “Theoretical, numerical and experimental study of geometrical parameters that affect anisotropy measurements in polarization-resolved SHG microscopy,” Opt. Express 23(7), 9313–9328 (2015). 10.1364/OE.23.009313 25968762

[31] Boothe T. Hilbert L. Heide M. et al., “A tunable refractive index matching medium for live imaging cells, tissues and model organisms,” eLife 6, e27240 (2017). 10.7554/eLife.27240 28708059 PMC5582871

[32] Dubois A. Levecq O. Azimani H. et al., “Line-field confocal time-domain optical coherence tomography with dynamic focusing,” Opt. Express 26(26), 33534–33542 (2018). 10.1364/OE.26.033534 30650800

[33] Ogien J. Levecq O. Azimani H. et al., “Dual-mode line-field confocal optical coherence tomography for ultrahigh-resolution vertical and horizontal section imaging of human skin in vivo,” Biomed. Opt. Express 11(3), 1327–1335 (2020). 10.1364/BOE.385303 32206413 PMC7075601

[34] Wasik V. Refregier P. Roche M. et al., “Precision of polarization-resolved second harmonic generation microscopy limited by photon noise for samples with cylindrical symmetry,” J. Opt. Soc. Am. A 32(8), 1437–1445 (2015). 10.1364/Josaa.32.001437 26367286

[35] Mahou P. Malkinson G. Chaudan E. et al., “Metrology of Multiphoton Microscopes Using Second Harmonic Generation Nanoprobes,” Small 13(42), 13 (2017). 10.1002/smll.201701442 28926684

[36] Born M. Wolf E., *Principles of Optics* (7th edition) (Cambridge University press, 1999).

[37] Demmerle J. Innocent C. North A. J. et al., “Strategic and practical guidelines for successful structured illumination microscopy,” Nat. Protoc. 12(5), 988–1010 (2017). 10.1038/nprot.2017.019 28406496

[38] Patel S. Tutchenko L., “The refractive index of the human cornea: A review,” Cont. Lens Anterior Eye 42(5), 575–580 (2019). 10.1016/j.clae.2019.04.018 31064697

[39] Yoo H. W. van Royen M. E. van Cappellen W. A. et al., “Automated spherical aberration correction in scanning confocal microscopy,” Rev. Sci. Instrum. 85(12), 123706 (2014). 10.1063/1.4904370 25554300

[40] Knighton R. W. Huang X. R. Cavuoto L. A., “Corneal birefringence mapped by scanning laser polarimetry,” Opt. Express 16(18), 13738–13751 (2008). 10.1364/oe.16.013738 18772985

[41] Gusachenko I. Schanne-Klein M.-C., “Numerical simulation of polarization-resolved second harmonic microscopy in birefringent media,” Phys. Rev. A 88(5), 053811 (2013). 10.1103/PhysRevA.88.053811

[42] Gusachenko I. Goulam Houssen Y. Tran V. et al., “Polarization-resolved second-harmonic microscopy in tendon upon mechanical stretching,” Biophys. J. 102(9), 2220–2229 (2012). 10.1016/j.bpj.2012.03.068 22824287 PMC3341536

[43] Tuer A. E. Akens M. K. Krouglov S. et al., “Hierarchical Model of Fibrillar Collagen Organization for Interpreting the Second-Order Susceptibility Tensors in Biological Tissue,” Biophys. J. 103, 2093–2105 (2012). 10.1016/j.bpj.2012.10.019 23200043 PMC3512050

[44] Duboisset J. Ait-Belkacem D. Roche M. et al., “Generic model of the molecular orientational distribution probed by polarization-resolved second-harmonic generation,” Phys. Rev. A 85(4), 043829 (2012). 10.1103/Physreva.85.043829

[45] Asadipour B. Beaurepaire E. Zhang X. et al., “Modelling and predicting second harmonic generation from protein molecular structure,” Phys. Rev. X 14(1), 011038 (2024). 10.1103/PhysRevX.14.011038

[46] Alizadeh M. Merino D. Lombardo G. et al., “Identifying crossing collagen fibers in human corneal tissues using pSHG images,” Biomed. Opt. Express 10(8), 3875–3888 (2019). 10.1364/BOE.10.003875 31452981 PMC6701537

[47] Tsafas V. Giouroukou K. Kounakis K. et al., “Monitoring aging-associated structural alterations in Caenorhabditis elegans striated muscles via polarization-dependent second-harmonic generation measurements,” J. Biophotonics 14(12), e202100173 (2021). 10.1002/jbio.202100173 34405541

[48] Ambekar R. Lau T. Y. Walsh M. et al., “Quantifying collagen structure in breast biopsies using second-harmonic generation imaging,” Biomed. Opt. Express 3(9), 2021–2035 (2012). 10.1364/BOE.3.002021 23024898 PMC3447546

[49] Mercatelli R. Mattana S. Capozzoli L. et al., “Morpho-mechanics of human collagen superstructures revealed by all-optical correlative micro-spectroscopies,” Commun. Biol. 2(1), 117 (2019). 10.1038/s42003-019-0357-y 30937399 PMC6435656

[50] Jeon H. Harvey M. Cisek R. et al., “Characterization of pathological stomach tissue using polarization-sensitive second harmonic generation microscopy,” Biomed. Opt. Express 14(10), 5376–5391 (2023). 10.1364/BOE.500335 37854565 PMC10581783

[51] Zheng T. Pendleton E. G. Barrow R. P. et al., “Spatial polarimetric second harmonic generation evaluation of collagen in a hypophosphatasia mouse model,” Biomed. Opt. Express 15(12), 6940–6956 (2024). 10.1364/BOE.529428 39679410 PMC11640570

[52] Montanino A. Gizzi A. Vasta M. et al., “Modeling the biomechanics of the human cornea accounting for local variations of the collagen fibril architecture,” Z. Angew. Math. Mech. 98(12), 2122–2134 (2018). 10.1002/zamm.201700293

[53] Giraudet C. Diaz J. Le Tallec P. et al., “Multiscale mechanical model based on patient-specific geometry: Application to early keratoconus development,” J. Mech. Behav. Biomed. Mater. 129, 105121 (2022). 10.1016/j.jmbbm.2022.105121 35290851

