# Supplementary material for: In situ determination and matching of the refractive index of the human cornea to improve polarization-resolved SHG imaging in depth
Source: Biomed Opt Express. 2025 Jul 18;16(8):3270–82. doi: 10.1364/BOE.564209 (PMC12339295; doi:10.1364/BOE.564209)
Supplement: Supplementary file 1 [file boe-16-8-3270-s001.pdf]

Supplemental Document

Biomedical Optics EXPRESS

In situ determination and matching of the refractive index of the human cornea to improve polarization-resolved SHG imaging in depth: supplement

PONCIA NYEMBO KASONGO,[1] PIERRE MAHOU,[1] JEAN-MARC SINTÈS,[1] GAËL LATOUR,[1,2,†] AND MARIE-CLAIRE SCHANNE-KLEIN[1,†,*]

[1] *Laboratory for Optics and Biosciences (LOB), École Polytechnique, CNRS, Inserm, Institut Polytechnique de Paris, 91120 Palaiseau, France*
[2] *Université Paris-Saclay, 91190 Gif-sur-Yvette, France*
[†] *These authors contributed equally.*
[*] *marie-claire.schanne-klein@polytechnique.edu*

This supplement published with Optica Publishing Group on 18 July 2025 by The Authors under the terms of the Creative Commons Attribution 4.0 License in the format provided by the authors and unedited. Further distribution of this work must maintain attribution to the author(s) and the published article's title, journal citation, and DOI.

Supplement DOI: https://doi.org/10.6084/m9.figshare.29529080

Parent Article DOI: https://doi.org/10.1364/BOE.564209

In situ determination and matching of the refractive index of the Human cornea to improve polarization-resolved SHG imaging in depth: supplemental document.

PONCIA NYEMBO KASONGO,[1] PIERRE MAHOU,[1] JEAN-MARC SINTÈS,[1] GAËL LATOUR,[1,2,†] AND MARIE-CLAIRE SCHANNE-KLEIN[1,†,*]

[1]*Laboratory for Optics and Biosciences (LOB), École Polytechnique, CNRS, Inserm, Institut Polytechnique de Paris, 91120, Palaiseau, France*
[2]*Université Paris-Saclay, 91190 Gif-sur-Yvette, France*
[†]*These authors contributed equally.*
**marie-claire.schanne-klein@polytechnique.edu*

Cornea #	Age	Gender	Post-mortem interval	Thickness (µm)	Reason of rejection from transplantation
1	59	M	< 24 hours	610	donor positive serology
2	69	F	< 24 hours	480	low endothelial quality
3	77	F	< 24 hours	600	low endothelial quality
4	59	M	< 24 hours	450	donor positive serology
5	71	M	< 24 hours	430	low endothelial quality
6	85	M	< 24 hours	540	donor positive serology
7	58	M	< 24 hours	570	low endothelial quality
8	85	F	< 24 hours	550	low endothelial quality

Sup. Table ST1: Detailed information about the corneas under study. All of the corneas under study exhibit good conservation.

Cornea #	Mean R^2			Mean number of valid voxels (%)		
	Config (i)	Config (ii)	Config (iii)	Config (i)	Config (ii)	Config (iii)
1	0.59	0.58	0.72	28	32	53
2	0.77	0.75	0.76	59	56	59
3	0.69	0.66	0.7	44	36	46
4	0.77	0.77	0.8	57	56	61
5	--	--	---	--	--	--
6	--	0.57	0.78	--	29	63
7	0.61	0.57	0.69	31	26	46
8	0.68	0.66	0.73	51	47	57
**mean**	**0.69**	**0.67**	**0.74**	**45**	**40**	**55**
**SD**	**0.08**	**0.08**	**0.04**	**13**	**13**	**7**

Sup. Table ST2: Mean R^2 and mean number of valid voxels in the posterior half of the corneas under study in the 3 configurations: (i) Without index-matching, $n_{collar} = n_{im} = 1.33$, (ii) Without index-matching, $n_{collar} = 1.36$, $n_{im} = 1.33$, (iii) With index-matching, $n_{collar} = n_{im} = n_{cor} = 1.38$. SD means Standard Deviation.

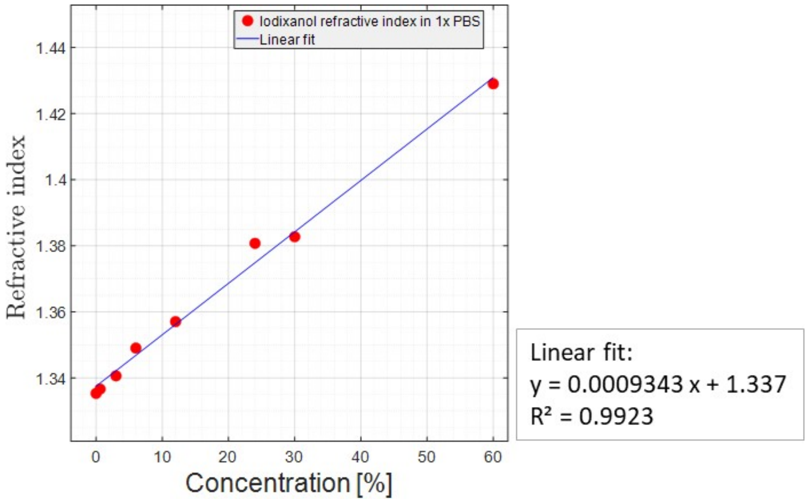

Fig. S1: Calibration of the refractive index as a function of the concentration of an iodixanol solution in PBS. The refractive index is measured using a Refracto30PX (Mettler-Toledo, Switzerland) at a wavelength of 589 nm and a temperature of 21° without any correction.

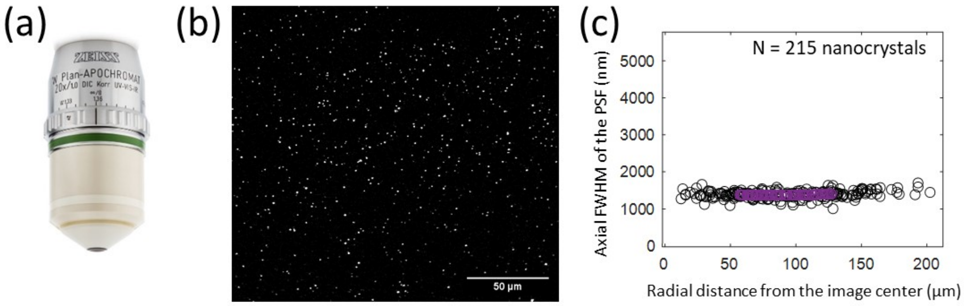

Fig. S2: Experimental resolution measurement. (a) Picture of the 20x, 1.0 NA water-immersion objective with a correction collar (W Plan-Apochromat 20×/1.0 Korr DIC, Zeiss). (b) Typical SHG image of KTP nanocrystals used for the resolution measurement. (c) Axial size (Full Width at Half Maximum) of each PSF computed from (b) as a function of the radial distance from the center of the field of view.

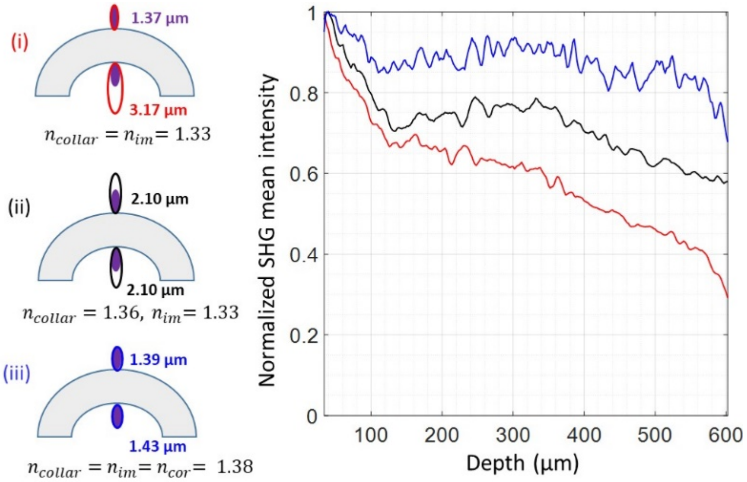

Fig. S3: Variation of the SHG signal along the depth of the cornea. The mean SHG signal in each 2D plane is plotted as a function of the depth within the cornea for the 3 imaging configurations: (i) red solid line: $n_{collar} = n_{im} = 1.33$, (ii) black solid line: $n_{collar} = 1.36, n_{im} = 1.33$ and (iii) blue solid line: $n_{collar} = n_{im} = 1.38$. All curves are normalized to their maximum value on the anterior part.

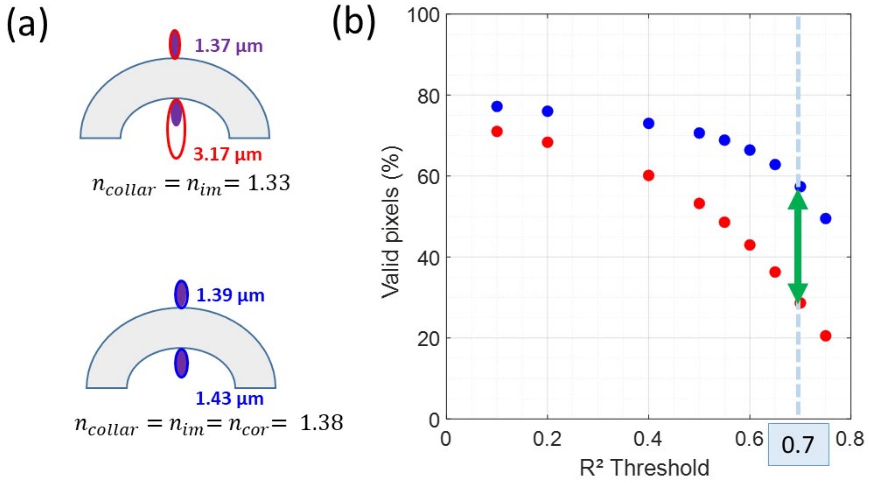

Fig. S4: Effect of the R^2 threshold value. (a) 2 imaging configurations under study: (red color) $n_{collar} = n_{im} = 1.33$, (blue color) $n_{collar} = n_{im} = 1.38$. (b) Variation of the percentage of valid pixels in the posterior stroma (400 to 500 µm deep) as a function of the R^2 threshold value. An R^2 threshold of 0.7 results in a 2.9-fold increase in the percentage of valid pixels for the refractive index matched configuration compared to the basic configuration with $n_{collar} = n_{im} = 1.33$.
